# Adverse event signal extraction from cancer patients’ narratives focusing on impact on their daily-life activities

**DOI:** 10.1038/s41598-023-42496-1

**Published:** 2023-09-19

**Authors:** Satoshi Nishioka, Masaki Asano, Shuntaro Yada, Eiji Aramaki, Hiroshi Yajima, Yuki Yanagisawa, Kyoko Sayama, Hayato Kizaki, Satoko Hori

**Affiliations:** 1 Division of Drug Informatics, Keio University Faculty of Pharmacy, Tokyo, Japan; 2 Nara Institute of Science and Technology, Nara, Japan; 3 Mediaid Corporation, Tokyo, Japan

**Keywords:** Disease prevention, Quality of life, Cancer

## Abstract

Adverse event (AE) management is important to improve anti-cancer treatment outcomes, but it is known that some AE signals can be missed during clinical visits. In particular, AEs that affect patients’ activities of daily living (ADL) need careful monitoring as they may require immediate medical intervention. This study aimed to build deep-learning (DL) models for extracting signals of AEs limiting ADL from patients’ narratives. The data source was blog posts written in Japanese by breast cancer patients. After pre-processing and annotation for AE signals, three DL models (BERT, ELECTRA, and T5) were trained and tested in three different approaches for AE signal identification. The performances of the trained models were evaluated in terms of precision, recall, and F1 scores. From 2,272 blog posts, 191 and 702 articles were identified as describing AEs limiting ADL or not limiting ADL, respectively. Among tested DL modes and approaches, T5 showed the best F1 scores to identify articles with AE limiting ADL or all AE: 0.557 and 0.811, respectively. The most frequent AE signals were “pain or numbness”, “fatigue” and “nausea”. Our results suggest that this AE monitoring scheme focusing on patients’ ADL has potential to reinforce current AE management provided by medical staff.

## Introduction

The incidence of cancers is rising worldwide, and the resulting clinical and economic burden is substantial^1^. Management of adverse events (AEs) during anti-cancer drug treatment directly affects patient compliance, and therefore can have a substantial impact on the outcome of anticancer treatment^2^. Early detection of the onset of such AEs and early mitigation are therefore important^3–6^.

In clinical outcome analysis for cancer therapeutics, the Common Terminology Criteria for AEs published by the National Cancer Institute (NCI-CTCAE) is the most widely used categorization tool to differentiate AEs by severity grade^7^. NCI-CTCAE ver.5.0 defines each AE category grade based on two axes; requirement of medical intervention and the magnitude of impact on activities of daily living (ADL). For example, Grade 2 events are defined based on the measure of “minimal, local or noninvasive intervention indicated” and “limiting age-appropriate instrumental ADL”. Although some AE grades are judgeable based on objective information such as laboratory test results, others depend solely on patients’ subjective symptoms. To capture those symptoms in a timely and accurate manner, a patient-oriented measure, the Patient-Reported Outcomes version of the CTCAE (PRO-CTCAE), has been developed^8^ and assessed in various studies for implementation in clinical settings^9–12^. Efforts to take account of patient-reported outcomes have recently attracted increasing interest, and are not limited to PRO-CTCAE^13–15^, but the methodology remains immature, and so multilateral approaches should be investigated to develop a fully effective patient-focused AE monitoring scheme.

AE complaints that are not reported to healthcare professionals during clinical visits are sometimes recorded during communications in internet patient communities^16^. Thus, we hypothesized that it might be feasible to capture early AE signals from patient community data and use them to enable earlier AE signal detection than would be possible by regular monitoring at the clinic. This would allow us to make use of material that has not previously been utilized for medical purposes to improve patient care. Indeed, some studies have already indicated that internet social communities can be useful for early detection of safety-related events^17–19^. This trend of early adverse event (AE) capture is consistent with other reports showing that patient-reported AEs are more frequent and occur earlier than physicians’ assessments^20–22^. Thus, early detection of AEs based on patient complaints in the internet community has the potential to be an effective approach for more proactive AE management by catching AE signals that occur outside hospitals, thereby improving cancer patients’ quality of life (QOL).

In recent years, it has been becoming a common approach to apply natural language processing (NLP) deep learning (DL) technology to various kinds of documents, including text data that requires contextual interpretations^23,24^. There have been a number of studies of medical applications of the technology, for example, to extract key information from electronic medical records^25–30^. The scope of research has also been expanding to patient-generated texts, such as articles posted in patient communities on the internet or on social media such as Twitter^31–34^. Our laboratory has also developed NLP models to extract specific AE events or patient worries from patient blogs^35,36^. Those studies showed that DL models could learn and extract specific signals for hand-foot syndrome or patients’ worries on physical matters, both of which contained patients’ real voices describing the impact on their daily lives. Thus, we thought it would also be feasible to utilize NLP tools to detect various kinds of AE signals from patient blogs focusing on those that limit ADL and which could be considered as equal to or higher than NCI-CTCAE Grade 2, possibly requiring immediate treatment interventions (e.g., interruption of primary cancer treatment or symptomatic therapies for AE management). There have been some previous studies on severity grading based on healthcare records^37–40^, but none has employed texts written by patients themselves. We considered that an AE signal extracting system focusing on impact on ADL could provide the basis for a practical AE monitoring scheme to efficiently detect AE signals indicating a need for professional medical support.

Here, we report the performance of this approach with NLP models using deep-leaning methods based on Bidirectional Encoder Representations from Transformers (BERT), ELECTRA and T5 to detect AE signals focusing on the impact on patients’ ADL as described in cancer patients’ blog articles.

## Results

### Dataset

**Figure 1:**
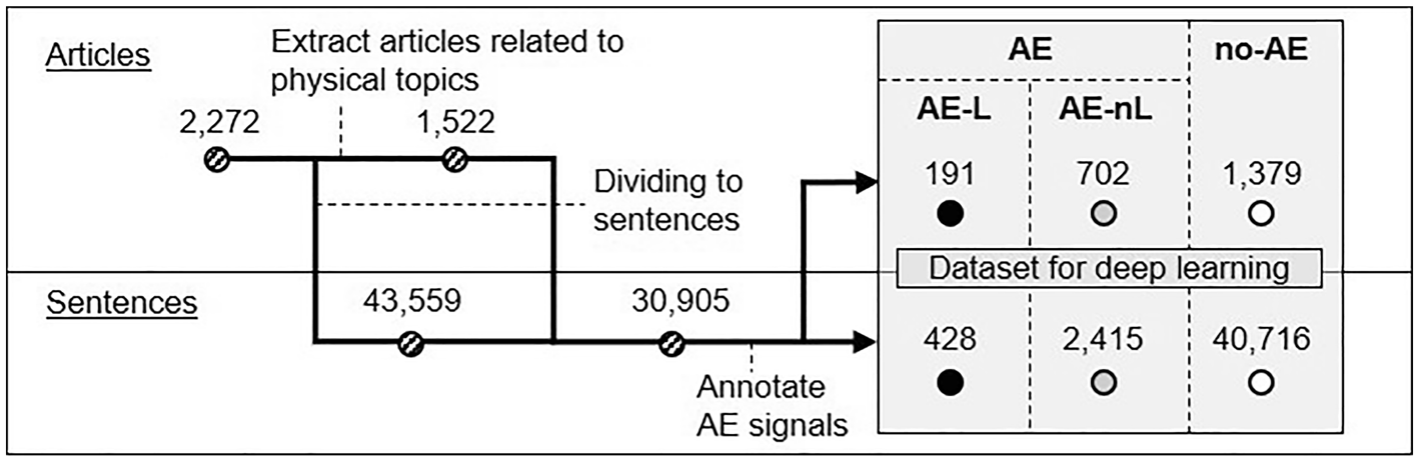
Annotation flow chart and dataset obtained for deep-learning.

According to the pre-defined annotation guideline for AE mentions (Table 1), two researchers annotated each sentence in the 1,522 posts on physical-related topics as AE limiting ADL (hereafter AE-L), AE not limiting ADL (hereafter AE-nL), or no-AE. The highest grade of sentences in an article was taken as the grade of the article. The kappa coefficient between the two annotators for 100 random posts was 0.819 for AE-L and 0.609 for all AE. As a result of the annotation process, 428 and 2,415 sentences, as well as 191 and 702 articles were identified as AE-L and AE-nL, respectively (Fig. 1). The sentences for AE-L and all AE accounted for 1.0% and 6.5%, respectively, of the 43,559 sentences written by breast cancer patients in the data set. At the article level, the percentages for AE-L and all AE were 8.4% and 39.3%, respectively, in the 2,272 posts.

**Table 1:** Annotation guideline for AE signals with impact on ADL.

	Definition
AE signal criteria	Any untoward medical occurrence in a patient regardless of the cause (i.e., includes not only potential drug side effects, but also events caused by primary disease, surgery or other physical events such as falling down). A sentence can be labeled as AE-L or AE-nL without clear AE mention if the sentence can be read out as an AE related comment considering the contexts before or after the sentence within the same blog post
- AE-L	- Sentences reveal clear limitations in ADL (i.e., describing specific limitations to patients’ ADL or physiological functions, or events not seen in a healthy condition and hindering ADL, such as vomiting or fever ≥ 38.0 degrees Celsius)
- AE-nL	- AE-mentioning sentences not falling into AE-L
	- Those touching on alopecia were all treated as AE-nL, because interpreting the magnitude of limitations to ADL from mentions of alopecia was infeasible in this study
Exclusion criteria (reason to exclude)	• Changes in mental state, such as worry or anxiety (because we could not judge whether they are AEs or just non-morbid daily emotional changes)
	• Mentions for recovery from AEs (when there are no on-going subjective symptoms)
	• Descriptions explaining about illness, future possibilities, or speculations (there are no actual events happening)
	• Reference to news articles etc. (there are no actual events happening)
	• Test results or findings without subjective symptoms (where no report is included from patients themselves)

The following table shows examples of actual mentions annotated as AE-L and AE-nL (Table 2). According to the pre-defined guideline, a majority of mentions for AE-L included specific negative impacts on patients’ ADL (e.g., “can't hold a pen firmly”, or “I can't even sit in front of the computer”). The other AE-L mentions described pre-defined events like vomiting or fever ≥ 38 degrees C, which would clearly impact on ADL. On the other hand, mentions for AE-nL included broad expressions about poor condition, but which were read as indicating light symptoms or from which the impact on ADL could not be determined in the absence of further information. Regardless of grade, lack of objective information was observed in many mentions (e.g., subjective terms, or the cause ascribed to the events). Patients’ original expressions, such as onomatopoeic ones, were another feature of the text generated by patients themselves.

**Table 2:** Examples of mentions annotated as AE-L and AE-nL.

	Japanese (original)	English (translated for reference)
AE-L	私は体調が悪くて起きてられずにダウンです。	I’ve been down sick and unable to stay awake
	昨夜38度台の熱が続いていた。	I had a fever of 38 degree C last night
	トイレに立ったら、激しい嘔吐に襲われた。	When I stood up to go to the bathroom I was attacked by severe vomiting
	夕ご飯はほとんど食べられない。	I could hardly eat dinner
	昨日は一睡もしていない。	I didn't sleep a wink last night
	インフルエンザの痛み × 10くらい。	Had ten lots of flu sore
	今月から生理が来なくなっている	I haven't had my period this month
	口の中、顔面もビリビリする・・・ボールペンがしっかり持てない。	Mouth and face are both numb (BIRI-BIRI*)…..can't hold a pen firmly
	PCに向かうのも無理。	I can't even sit in front of the computer
	人に逢えなくなった。	I can't see people anymore
	電話にも出なくなった。	I can't answer the phone anymore
AE-nL	まだ全身の倦怠感でだるい。	Still sluggish with general malaise
	鼻水垂れている。	Runny nose dripping
	麻酔が切れてくると痛い痛い。	Aches as the anesthesia wears off
	昨夜は吐き気はしたが、朝まで結構眠れた。	I had nausea last night, but slept well until morning
	足のしびれで泣いた。	I cried because of numbness in my feet
	抜けるものは抜けます(笑)	Can't stop the hairs from falling out, lol
	口の中がボコボコした感じになった。	My mouth felt bumpy (BOKO-BOKO*)
	キタキタキター!!何がって、副作用が…。	It’s coming!!!! What is side effect…

Asterisk mark (*) indicates onomatopoeic expression in Japanese.

### Performance of trained DL models

#### Straightforward approach

At the sentence level, BERT and ELECTRA were used to identify all AE or AD-L mentions from 43,559 sentences. For comparison purpose to a traditional model without DL feature, multinomial Naive Bayes model was also used in the same tasks. ELECTRA showed slightly better F1 score than BERT for all AE (0.640 versus 0.589, *p* < 0.05), while both showed similar F1 scores for AE-L (0.301 and 0.299 for BERT and ELECTRA, respectively). It was also confirmed that the two DL models showed better performances compared to the multinomial Naive Bayes model for both all AE and AD-L (*p* < 0.05) (Table 3). Precision-recall (PR) curve and value of the area under the curve (AUC) for each DL model and task are shown in Supplementary Fig. S1.

**Table 3:** Performance scores at the sentence level in the straightforward approach.

	Precision	Recall	F1 score
All AE			
Multinomial naive bayes	0.591 (0.027)	0.336 (0.021)	0.428 (0.024)
BERT	0.596 (0.033)	0.585 (0.060)	0.589 (0.035)*
ELECTRA	0.641 (0.014)	0.641 (0.031)	0.640 (0.013)*^, †^
AE-L			
Multinomial naive bayes	0.227 (0.042)	0.095 (0.015)	0.133 (0.017)
BERT	0.221 (0.037)	0.486 (0.080)	0.301 (0.033)*
ELECTRA	0.215 (0.034)	0.498 (0.079)	0.299 (0.038)*

Each number indicates “mean (s.d.)” from n = 5 with different random state setting. Asterisk and dagger marks indicate statistical significance in F1 scores between evaluated models after Bonferroni correction; * *p* < 0.05 for BERT or ELECTRA versus Multinomial Naive Bayes, † *p* < 0.05 for ELECTRA versus BERT.

At the article level, BERT, ELECTRA and T5, as well as multinomial Naive Bayes, were used to extract articles with all AE or AE-L mentions among 2,272 articles. For BERT, ELECTRA and multinomial Naive Bayes, the highest grade in predictions for sentences in a given article was noted as the grade of the article. The performance scores at this article level are shown in Table 4. Among the three tested DL models, T5 showed the best F1 score in both all AE and AE-L tasks, 0.811 and 0.557, respectively, with significant difference from BERT and ELECTRA (*p* < 0.05). ELECTRA and BERT showed similar F1 scores for both all AE and AE-L. All the three DL models outperformed the multinomial Naive Bayes model in both all AE and AE-L tasks with significant difference (*p* < 0.05). PR curve and AUC value for each DL model and task are shown in Supplementary Fig. S2.

**Table 4:** Performance scores at the article level in the straightforward approach.

	Precision	Recall	F1 score
All AE			
Multinomial naive bayes	0.671 (0.026)	0.448 (0.031)	0.537 (0.028)
BERT	0.666 (0.038)	0.700 (0.073)	0.680 (0.034)*
ELECTRA	0.713 (0.026)	0.749 (0.058)	0.728 (0.014)*
T5	0.798 (0.050)	0.827 (0.029)	0.811 (0.016)*^, †^
AE-L			
Multinomial naive bayes	0.293 (0.061)	0.144 (0.033)	0.192 (0.037)
BERT	0.281 (0.044)	0.648 (0.073)	0.388 (0.034)*
ELECTRA	0.263 (0.036)	0.626 (0.034)	0.368 (0.032)*
T5	0.661 (0.039)	0.484 (0.044)	0.557 (0.022)*^, †^

Each number indicates “mean (s.d.)” from n = 5 with different random state setting. Asterisk and dagger marks indicate statistical significance in F1 scores between evaluated models after Bonferroni correction; * *p* < 0.05 for BERT, ELECTRA or T5 versus Multinomial Naive Bayes, † *p* < 0.05 for T5 versus BERT or ELECTRA.

#### Grading approach

Using BERT and ELECTRA, another approach to classify each sentence or article into three grades (i.e., AE-L, AD-nL or no-AE) was investigated. In this approach, we evaluated how DL models can learn the differences between the three grades. The results of macro average scores at the sentence and article levels are shown in the Appendix (Supplementary Table S1). The best F1 score was approximately 0.63 in ELECTRA at both the sentence and article levels. When looking at specific prediction results and performance scores for the ELECTRA model at the article level, the F1 scores for AE-L and AE-nL were 0.418 and 0.600, respectively (Supplementary Table S2).

#### Two-step approach

We hypothesized that removing noisy sentences (i.e., no AE-including mentions) would lead to more efficient learning for AE-L and AE signal features, and tried a two-step approach with combination of ELECTRA and T5. The performance scores for the AE-L article identification task, as well as the all AE sentence extraction in the 1st step, are shown in the Appendix (Supplementary Table S3). Contrary to our expectation, the best F1 score for AE-L articles in this approach was not improved compared to that in the straightforward approach: 0.351 versus 0.557.

### Features of AE symptoms

**Figure 2:**
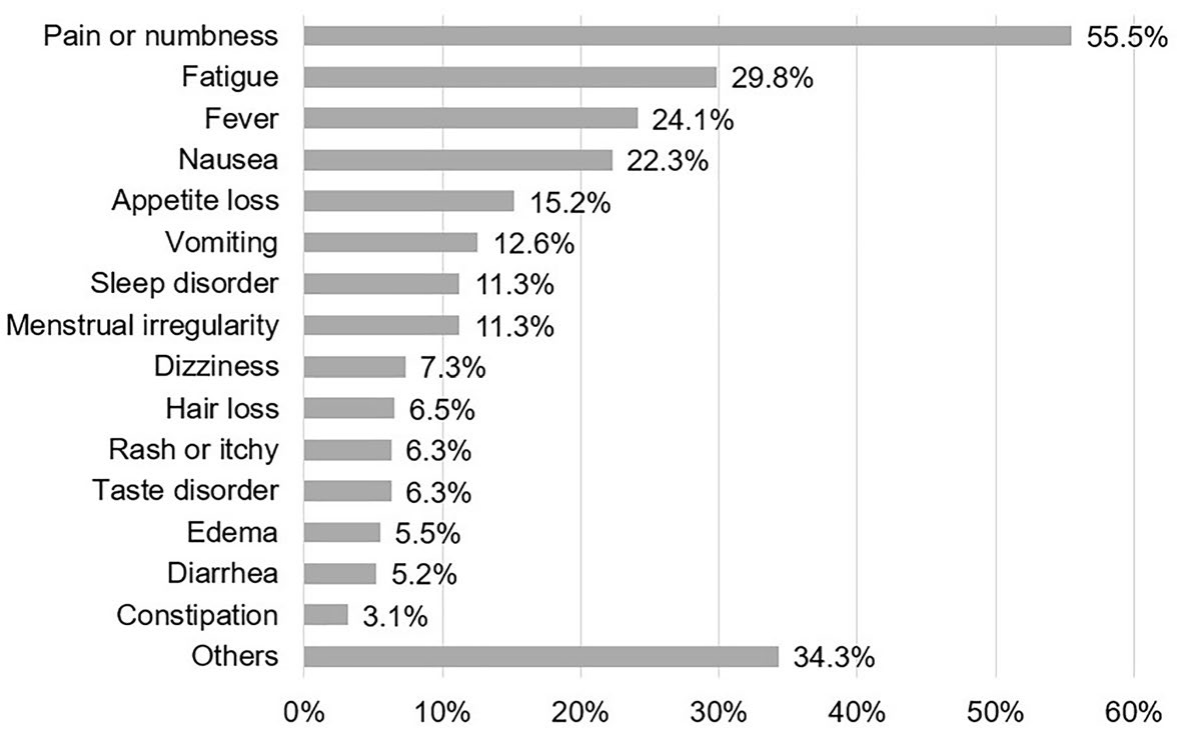
Percentages of AE signals by category in AE-L-containing articles. The percentage was calculated by dividing articles with AE-L signals in each category by all AE-L-containing articles (i.e., 191 articles).

To grasp the features of AE signals described in the blog posts, retrospective AE categorization was conducted for each article with AE mentions according to the pre-defined guideline (Supplementary Table S4). In the 191 articles with AE-L signals written by breast cancer patients, frequent AE categories (accounting over 20%) were “pain or numbness” (55.5%), “fatigue” (29.8%), “fever” (24.1%), and “nausea” (22.3%) (Fig. 2). When comparing the category proportions in the articles with AE-L signals to those in the 893 articles with all AE signals (Supplementary Fig. S3), similar trends were observed for “pain or numbness” and “fatigue”. On the other hand, “fever”, “nausea”, “appetite loss”, “vomiting”, “sleep disorder” and “menstrual irregularity” were more frequently described in the articles with AE-L (i.e., over 20% for “nausea” and “fever”, and over 10% for the rest). “Others” included other scattered symptoms including but not limited to “stomatitis” and “dyspnea”, or patients’ unique expressions that did not fall into other defined categories (e.g., “Feeling like I have a steel plate in my chest”).

## Discussion

In this study, we developed DL systems to extract AE signals from patients’ written texts, focusing on AE severity, i.e., the actual impact on their ADL. Previous work on distinguishing AE severity grades from healthcare-related documents has employed only hospital records or AE incident reports in a public database^37–40^. Here, we focused on the texts written by patients themselves, which are expected to include more direct expressions and descriptions of subjective symptoms. As it is known that patients often do not mention every single symptom in clinic visits^20–22,41^, our approach to directly capture AE signals from patients’ voices could contribute to more effective early AE signal detection. To elaborate, we expect two positive effects in clinical settings by applying this system to web patient communities or electronic patient diaries, including social media: one is to encourage patients to visit the clinic by alerting them to AE signals limiting ADL, and the other is to notify medical staff of potentially severe AE signals which may require immediate medical interventions. This automatic screening with DL models focusing on impact on ADL could cost-effectively highlight important AE signals that might be missed under current AE monitoring schemes relying on medical staff only, thus leading to earlier intervention for AEs and better anti-cancer treatment.

In our study, the best F1 scores for AE-L and all AE extractions at the article level were 0.557 and 0.811, respectively (Table 4), and we regard these results as positive. In connection with AE-L extraction, previous studies aimed at automatic severity grade classification achieved F1 scores of more than 0.8^38,39^. Direct comparison between those results and our study results, however, is not appropriate from the point of view of the substantial differences in study conditions, including the types of source documents (e.g., healthcare records containing clear severity information versus patient narratives without such information) and the severity grading scale (e.g., one study utilized health care records along with a harm scale in the Common Formats^39^). As is discussed below under “error analysis”, the F1 score of 0.557 for AE-L events would justify further assessment in a clinical pilot study, as the majority of the false positive cases were AE-nL events. As for all AE extraction regardless of impact on ADL, the best F1 score of 0.640 at the sentence level (Table 3) is only slightly lower than the result of a competitive shared task, Social Media Mining for Health Applications (SMM4H) workshop 2022, where the best F1 score for an AE extraction task from Twitter posts containing drug mentions was 0.698^34^. It seems likely that technical developments and adjustments will further improve the performance scores in the future. This AE mention extraction model at the sentence level would contribute to identifying the phrases that patients actually use to talk about AEs from the posted articles.

**Figure 3:**
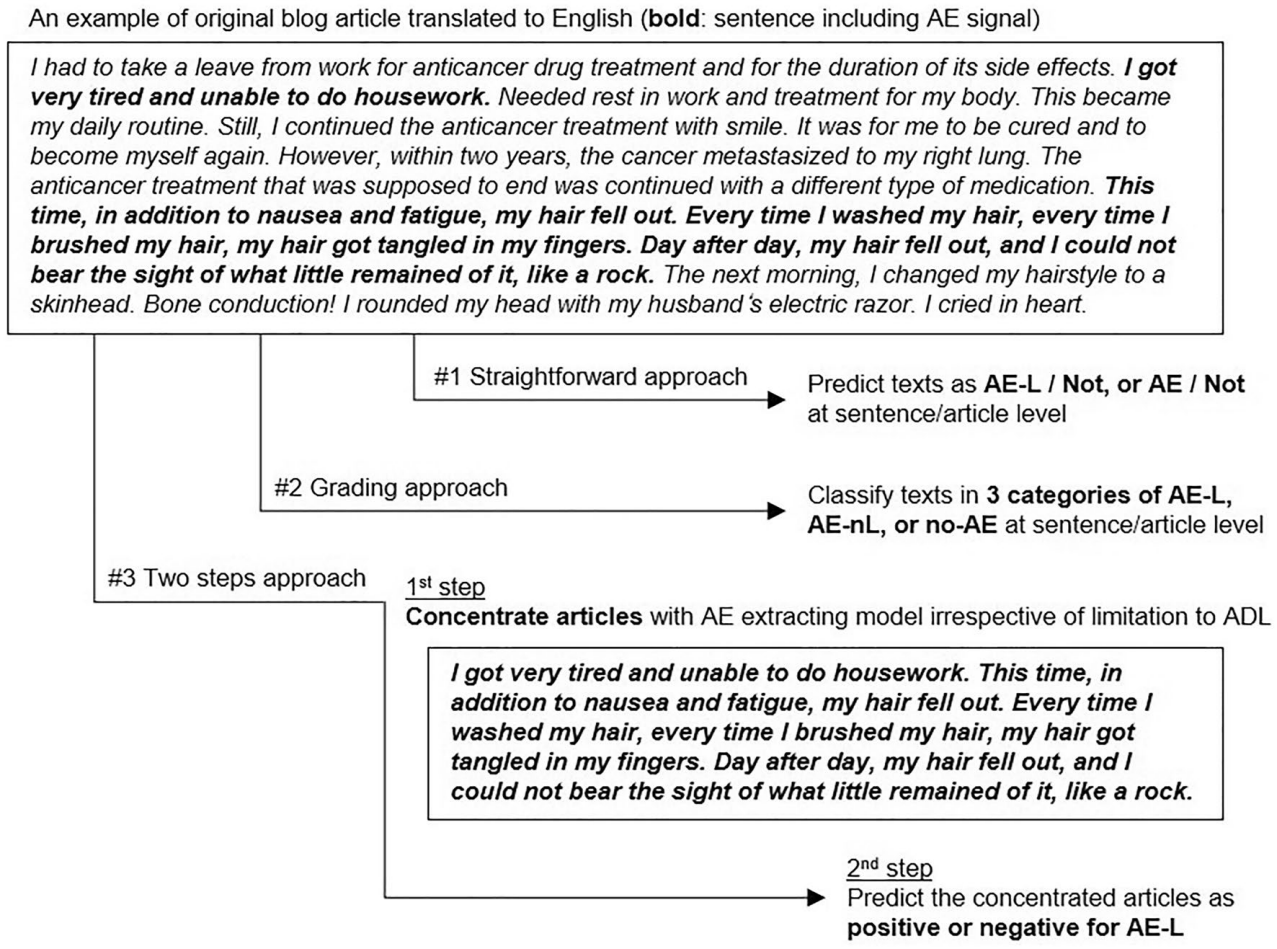
Three approaches for AE-L signal identification.

T5 showed the best F1 scores of 0.557 and 0.811 for AE-L and all AE, respectively, at the article level among the tested DL models in our study (Table 4). We consider that a major reason for the better scores in T5 than those in BERT and ELECTRA is the difference in input for training and prediction; T5 input all texts in each article at once, while BERT and ELECTRA input each separate sentence in each article. Thus, T5 could learn efficiently based on not only more information in one input, but also well-organized context in each original article compared to the other two DL models. Although we selected sentence-based input for BERT and ELECTRA due to the limitation of 512 tokens and the high volume of the source texts in the patient blog posts (Supplementary Fig. S4), article-based input should be considered for BERT and ELECTRA if the volume of source data permits. When comparing results for AD-L identification task at the article level among the three approaches (i.e., straightforward, grading and two steps), the straightforward approach outperformed the other 2 approaches. As for the grading approach, false positives from biased data to no-AE or AE-nL resulted in a lower F1 score of 0.418 for AE-L (Supplementary Table S2). Direct extraction of AE-L, i.e., the straightforward approach, would work better than the grading approach when focusing on only AE-L, while the latter could be useful to acquire AE signal distribution by severity with a single classification. When it comes to the two-step approach, the relatively low F1 scores of 0.334 to 0.351 (Supplementary Table S3) were not in line with our expectation, because we originally hypothesized that pre-processing to concentrate on blog posts with AE mentions would highlight the difference of features between AE-L and AE-nL. We retrospectively considered two causes for the unexpectedly low scores in the two-step approach: one was insufficient quality of the first-step concentration by all AE extraction, and the other was lack of context in each article. The former means that the all AE extraction models used as the first step in this study did not work well enough, as the Model 1 lost 26.5% of AE signals based on the recall of 0.735 or the Model 2 still included too many non-AE mentions based on the precision of 0.320 (i.e., more than twice of non-AE mentions than the true AE mentions) (Supplementary Table S3). The latter reason, lack of context, means that the context in original articles is lost when concentrating those with only AE mentions so that accurately reading-out the background behind each word or phrase could become harder for T5 (for example, as shown in Fig. 3, there was context in the original article, such as *“… However, within two years, the cancer metastasized to my right lung. The anticancer treatment that was supposed to end was continued with a different type of medication. This time, in addition to nausea and fatigue,* …”, while the context lost after removing non-AE mentions between AE mentions, leaving “***I got very tired and unable to do housework. This time, in addition to nausea and fatigue, …***” where information about cancer re-occurrence and re-challenge of anti-cancer treatment was lost). These may be the reasons why the two-step approach did not work effectively as we originally hypothesized.

We also retrospectively analyzed the characteristics of AE mentions posted in the patients’ blogs (Fig. 2). The most frequent AE signals of “pain or numbness”, “fatigue” and “nausea” in all AE are consistent with the trend observed in previous research; more frequent or more severe events were reported for fatigue, nausea and pain in patient-reported assessments compared to clinician-reported ones^20–22^. It seems to be clear that those symptoms need careful monitoring beyond clinical visits. On the other hand, relatively small proportions of incidents were observed for “diarrhea”, “vomiting”, or “constipation”, in contrast to past research. This discrepancy may be due to patients’ desire to avoid such dirty topics in their blog posts. When comparing AE-L (Fig. 2) with all AE (Supplementary Fig. S3), the proportions of “fever”, “nausea”, “appetite loss”, “vomiting”, “sleep disorder” and “menstrual irregularity” increased to more than 10%. These symptoms could be meaningful signs to identify patients experiencing AE-L AE signals based on their narratives.

Finally, we conducted error analysis. Most false positives by our T5 model for AE-L were those with AE-nL mentions. Considering that such apparently false positive instances could harbor potential AE-L events that were not clearly expressed in the blog texts, such false positives might be worthy of further assessment by medical staff in real clinical practices. Some articles without any AE mentions were also inaccurately predicted as AE-L; these false positives tended to have one or both the following characteristics: describing impaired ADL because of non-AE events (e.g., too much walking) or describing the time course of clinical treatment in detail. The former case is in line with our focus on negative impact on ADL, although it will be necessary to improve our models for the AE monitoring purpose by distinguishing AE and non-AE as the cause. In the latter case, the authors of those articles might be extremely sensitive to physical changes in their body, which suggests that careful interviews might detect hidden AE signals from them. To investigate how those false positive instances might hinder implementation in the real world, as well as to establish how high an F1 score is required for practical implementation, a prospective study with the AE signal detection system will be required.

There were three limitations associated with the training dataset for our study: the first is the relatively small dataset for DL (i.e., larger training datasets could improve the performance scores); the second is lack of diagnostic information from medical doctors (i.e., it was infeasible to validate identified AE signals or grades with assessment from the perspective of a medical expert in this study); the third is the shortage of objective information to identify specific AE signals in patients’ written texts (e.g., the definitions of AE categories were broad, such as “pain or numbness” or there were uncategorized symptoms included in “others”). To overcome these limitations, collecting a larger training dataset or conducting a prospective study linked with medical records would be desirable. Another limitation is that the present study was not designed to evaluate the uncertainty of each DL model. The measurement of uncertainties in DL prediction models is an important factor in model development^42^, and further work will be needed on this issue for practical implementation.

In conclusion, our results support the feasibility of a novel approach to detect AE signals for AE-L events from patient narratives. Such events might need immediate medical intervention, but could be missed under current AE monitoring systems supported by medical staff only. Our results suggest that the use of DL models to detect AE signals according to severity grade in texts written by cancer patients might result in earlier AE signal detection, enabling earlier intervention and improving patients’ QOL.

## Methods

### Data source

Blog posts written in Japanese by breast cancer patients in a web community, LifePalette^43^, were utilized for this study. Life Palette is an internet patient community in Japan. In the web community, patients can freely record diaries or experiences in their daily lives with illness, and many cancer patients have posted on it. Patients can also expand their personal network and exchange messages with each other in the web community, if they find someone they want to connect with (e.g., patients with the same illness, side effects, or concerns). The data source consisted of 13,570 articles written by 289 users, posted on Life Palette from Mar 6^th^, 2008, to Nov 20^th^, 2014. A total of 2,272 breast cancer posts were retrospectively extracted for this study, excluding drafts and duplicates; this was the same data set analyzed in the work by Watanabe et al^36^.

From the 2,272 posts, those which include at least one complaint about an AE were extracted through pre-processing and annotation of AE mentions.

### Pre-processing

The 2,272 posts were divided into sentences by using the publicly available open-source “ja_sentence_segmenter”^44^. This resulted in 43,559 sentences after the removal of duplicated sentences. In parallel, 1,522 posts mentioning physical related topics among the 2,272 posts were identified through output from the earlier work by Watanabe et al^36^. The 1,522 posts were also divided into sentences by the segmenter, which afforded 30,905 sentences. This process is illustrated in Fig. 1 in the "Results" Section.

For the later stage of training of DL models, under-sampling was implemented for negative label data to correct training bias when the proportion of positive label data was less than 5% in the training dataset.

### Annotation

With the 30,905 sentences, annotation was conducted by two independent annotators (SN and MA) based on pre-defined guidelines for AE mentions (Table 1). The guideline was developed by the primary author (SN) to distinguish those limiting ADL or not in line with the grade definition in NCI-CTCAE ver.5.0^7^, although some adjustments were required for this study, such as exclusion of mental state changes or only test results.

The validity and reproducibility of the annotation guideline (Table 1) were ensured through guideline refinement based on measurement of the kappa coefficient^45^. Initially, the two annotators independently annotated 50 randomly extracted posts according to the first version of the guideline, and the kappa coefficient between them was calculated. As the first kappa coefficient value was insufficient for AE-L posts (i.e., < 0.3), the annotation guideline was refined to increase the clarity of the AE category definitions. Afterward, another 50 randomly extracted posts were annotated by the two annotators according to the updated guideline. At this point, the annotation guideline was finalized because the kappa coefficient was confirmed to be sufficiently large for AE-L posts (i.e., > 0.8). To ensure the guideline validity, another 50 randomly extracted posts were annotated per the same guideline and the final concordance level was confirmed by calculation of the kappa coefficient from the total of 100 randomly extracted posts, which consisted of 2,334 sentences.

Thereafter, one of the annotators (SN) completed annotation for the remaining sentences according to the finalized annotation guideline (Table 1). Finally, all sentences were annotated as AE-L, AE-nL, or no-AE.

### Deep learning models

The DL methods used in this study were BERT^46^, ELECTRA^47^, and T5^48^; these were selected as they have recently shown high performance in a number of studies^34,49^. For BERT and ELECTRA, sentence level data was utilized as the training or test data set to limit the length of input less than 512 tokens, as 23.4% of the source blog posts exceeded 512 tokens (Supplementary Fig. S4). For T5, the original article data was used up to 2,000 tokens for both training and test steps. The reason for the upper limit of 2,000 tokens was to reduce the operational burden for the training, based on confirmation that there would be little impact on study results with the limit setting (Supplementary Fig. S4). To efficiently learn Japanese word embeddings, publicly open pre-trained models were utilized for each DL method^50–52^. Using a given random state setting, 80% of the annotated data was used as a training dataset for additional training, and the remaining 20% was used as a test dataset for performance evaluation. For the purpose to compare DL models with a traditional model not using DL feature, document classification model based on multinomial Naive Bayes^53^ was also evaluated at both sentence and article level with the same training and test dataset.

### Approaches to identify AE limiting ADL

The main purpose of this study is to extract AE signals from patient narratives focusing on AE-L. To achieve this, we tried three approaches: #1 simply predict AE-L sentences or articles (called the “straightforward approach”); #2 classify sentences or articles in three grades of AE-L, AE-nL and no-AE (called the “grading approach”); and #3 firstly filter out non-AE mentions from individual articles then identify AE-L from the dense articles with AE mentions (called the “two-step approach”) (Fig. 3). For the straightforward approach, we also evaluated prediction models for all AE signals (i.e., AE-L or not, plus AE-nL or not) for comparison with previous research^34^, utilizing this as the 1st step for the two-step approach.

### Evaluation

The performance of each model to identify AE signals was evaluated in terms of precision, recall and F1 score. We evaluated our trained models with the parameters at the sentence level for BERT, ELECTRA and multinomial Naive Bayes, and at the article level for all methods including T5.

$$Precision=\frac{\#\; of \;True \;Positive}{\#\; of\; True \;Positive+False\; Positive}$$

$$Recall= \frac{\#\; of\; True\; Positive}{\# \;of\; True \;Positive+False\; Negative}$$

$${F}_{1}\; score=\frac{ 2*precision*recall}{precision+recall}$$

After we identified that the straightforward approach performed better than the other 2 approaches, five times of experiments were conducted with different random state settings for each model in the straightforward approach respectively, then mean value and standard deviations were calculated for each score. For evaluation of significant difference in F1 scores between models, paired t-test was adopted because each experiment used the same dataset across models which was prepared with different random state settings. P value for a two-tailed test was evaluated (i.e., *p* < 0.05 or not) after Bonferroni correction for the multiple-comparison. In addition, PR curve was created for individual representative experiments using seamless decision threshold from 0 to 1, along with the value of AUC, to interpret average model performance.

### AE signal categorization

All of the annotated posts were retrospectively categorized into type of AE symptoms. The guideline for the categorization was created by the primary author (SN) to assess the features of AE mentions described in the data source (Supplementary Table S4), referring to previous research^21,22^ and PRO-CTCAE^8^. Because of the limitation of lacking information in patient-generated texts, the category “pain or numbness” was defined broadly regardless of the cause or body site. According to the guideline, two independent researchers (YY and KS) connected all blog articles including AE mentions with multi-label categories.

### Ethical approval

In accordance with Life Palette's terms of service, agreements from all contributing users for secondary anonymous use of blog posts by a third party for research purposes were obtained on their individual starting dates of service use, by checking the Agree button on the website. Our laboratory (Keio University) obtained the blog post data for research purposes from the operating company, Mediaid Corporation, based on a joint research agreement. This study was carried out with their anonymized data, following approval by the ethics committee of the Keio University Faculty of Pharmacy (the latest approval No. 230126–1), in accordance with relevant guidelines and regulations or the Declaration of Helsinki. Informed consent specific for this study was waived due to the retrospective observational design of the study based on the approval by the ethics committee of the Keio University Faculty of Pharmacy.

### Supplementary Information

Supplementary Information.

## Data Availability

The data consisting of blog articles in the study are available from Mediaid Corporation upon reasonable request.
