# Supplementary material for: Adverse event signal extraction from cancer patients’ narratives focusing on impact on their daily-life activities
Source: Sci Rep. 2023 Sep 19;13:15516. doi: 10.1038/s41598-023-42496-1 (PMC10509234; doi:10.1038/s41598-023-42496-1)

Title

Adverse event signal extraction from cancer patients' narratives focusing on impact on their daily-life activities

Authors

Satoshi Nishioka[1], Masaki Asano[1], Shuntaro Yada[2], Eiji Aramaki[2], Hiroshi Yajima[3], Yuki Yanagisawa[1], Kyoko Sayama[1], Hayato Kizaki[1] & Satoko Hori[1,*]

1 Division of Drug Informatics, Keio University Faculty of Pharmacy, Tokyo, Japan
2 Nara Institute of Science and Technology, Nara, Japan
3 Mediaid Corporation, Tokyo, Japan
* satokoh@keio.jp

Supplementary Table S1. Performance scores (macro average) in the grading approach

	Precision	Recall	F1
**Sentence level**			
BERT	0.497	0.768	0.555
ELECTRA	0.571	0.763	0.634
**Article level**			
BERT	0.512	0.700	0.524
ELECTRA	0.587	0.755	0.632

Supplementary Table S2. Prediction results with performance scores for ELECTRA at the article level in the grading approach

		Predicted label			Precision	Recall	F1
		no-AE	AE-nL	AE-L			
True label	no-AE	1285	259	71	0.796	0.982	0.879
	AE-nL	23	253	32	0.821	0.472	0.600
	AE-L	1	24	46	0.648	0.309	0.418

Supplementary Table S3. Performance scores for the 1st step (All AE sentence extraction) and 2nd step (AE-L article identification) in the two-step approach

	Precision	Recall	F1
**1st step by ELECTRA**			
Model 1 – without under-sampling	0.574	0.735	0.645
Model 2 –with under-sampling (positive : negative = 1:1 in training dataset)	0.320	0.924	0.476
**2nd step by T5**			
Following to model 1	0.350	0.320	0.334
Following to model 2	0.500	0.270	0.351

Supplementary Table S4. Guideline for AE symptom categorization

Category	Definition	Examples in Japanese (original)	Examples translated to English (for reference)
**Fatigue**	All kinds of expressions indicating general bad condition	疲れた, だるい, 具合わるい, へとへと, ダウン, 何もできない	Tired, Lazy, Uncomfortable, HETO-HETO*, Down, Unable to do anything
**Nausea**	Any expressions that indicate nausea happened	吐きそう、気持ち悪い, 胃がぐるぐる	Want to vomit, Feeling sick, Stomach upset
**Vomiting**	Mentions that clearly indicate vomiting occurrence	吐いた, 戻した, 食べたもの全部出た	Vomiting, Regurgitation, Everything I've eaten has come out
**Diarrhea**	Any expressions that can be read as diarrhea symptoms or more frequent defecation than normal	下痢, 便がゆるい,トイレから出られない	Diarrhea, Loose stools, Unable to leave the bathroom
**Constipation**	Mentions that indicate symptoms of constipation	便が出ない, お通じが悪い	No stool, Bad bowel movement
**Appetite loss**	Any expressions that can be read as decreased appetite, frequency or amount of meals	食欲がでない, 何も喉を通らない, ろくなものを食べてない	Loss of appetite, Don't want to eat anything, Cannot eat enough
**Pain or numbness**	All complaints of pain or numbness, regardless of cause or hurting sites (i.e., including but not limited to headache, arthralgia, vascular pain, stomatitis, pain due to primary tumor or surgery)	xxx が痛い, xxx が痺れる, ひりひり, ガンガン響く	Hurt somewhere, Numbing, HIRI-HIRI*, GAN-GAN*
**Rash or itchy**	All kinds of mentions of skin rashes, pimples, or itching that didn't fall under pain or numbness	かゆい, ぶつぶつができた, 吹き出物が気になる, つい引っ掻いてしまう	Itching, Bumps, Pimples, Scratching

**Hair loss**	Mentions that indicate hair loss happened	ごっそり抜ける, 起きたら枕がすごいことに, 頭を洗うたびに落ち込む, かつらを探さないと	It's falling out a lot, A pillow full of hair, Feeling depressed every time I wash my head, Need to find a wig
**Menstrual irregularity**	Any expression that could be read as menstrual irregularity	ついに生理が来なくなった, 数か月来ていない	I finally stopped having my period, Haven't had my period for months
**Fever**	Mentions that indicate fever more than ≥ 37.0 degrees C	発熱, 熱を測ったら 38 度	Fever, 38 degrees Celsius
**Taste disorder**	Mentions that indicate abnormal taste happened	味が分からない, 美味しくない, xxx を食べると気持ち悪い	Cannot taste anything, Doesn't taste good, Feel sick when I eat something
**Dizziness**	Any descriptions of dizziness symptoms that didn't fall under fatigue	頭がくらくら, ふらっとする	Lightheadedness, KURA-KURA*, Stagger
**Sleep disorder**	Any expressions that indicate insomnia or somnolence	眠れない, 夜何回も起きる, 眠くて日中何もできない	Cannot sleep, Waking up several times a night, Too sleepy to do anything during the day
**Edema**	Mentions that indicate edema occurrence	腫れている, ぱんぱん	Swollen, PAN-PAN*
**Others**	Those didn't fall under any of above	xxx ができない	Cannot do something

* Onomatopoeic expression in Japanese

Supplementary Figure S1. PR curve and AUC value for DL models at the sentence level in the straightforward approach

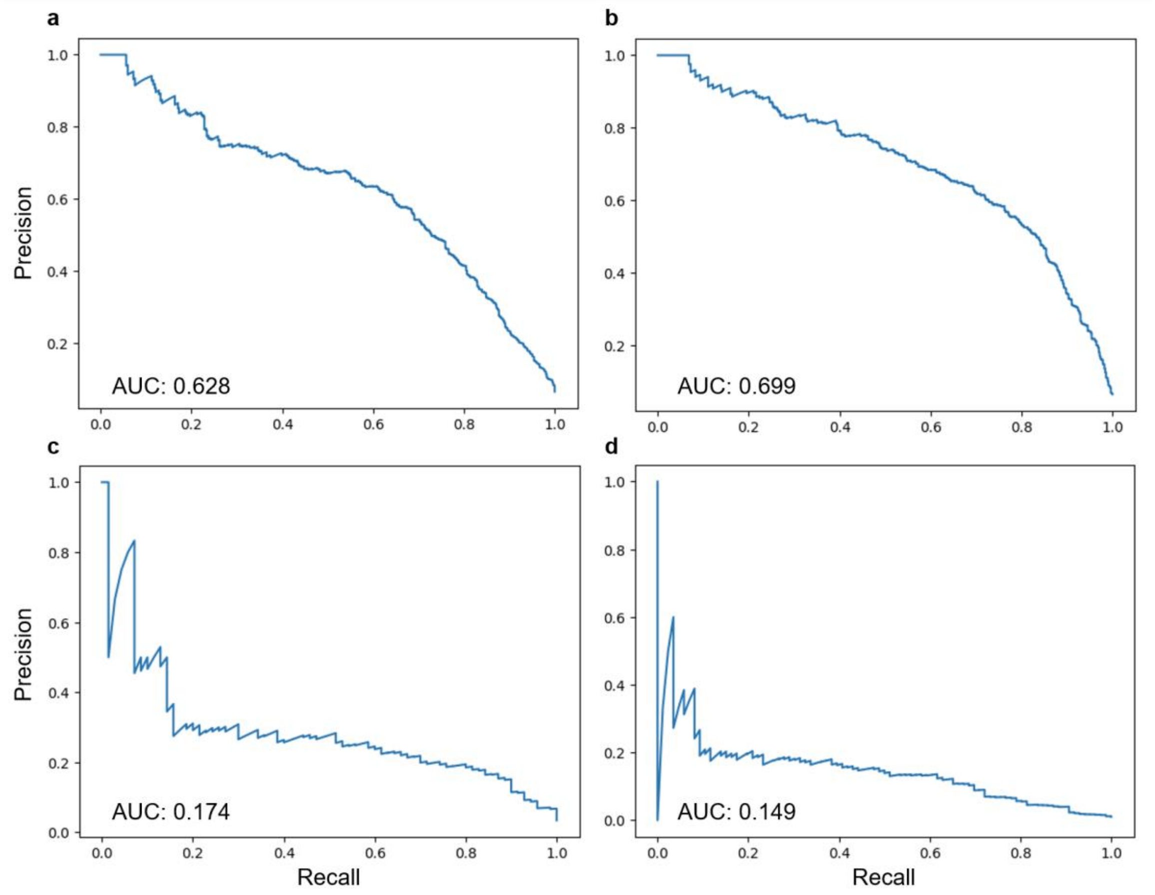

PR curve and AUC value at the sentence level in all AE task for BERT(a) and ELECTRA (b), as well as those in AE-L task for BERT(c) and ELECTRA (d).

Supplementary Figure S2. PR curve and AUC value for DL models at the article level in the straightforward approach

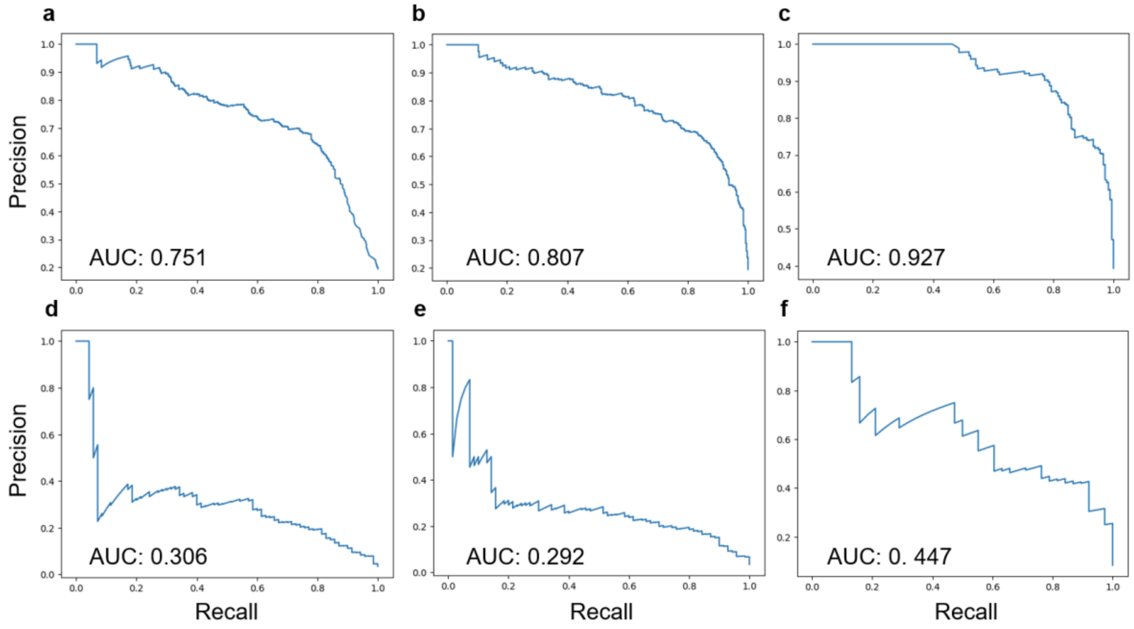

PR curve and AUC value at the article level in all AE task for BERT(a), ELECTRA (b) and T5 (c), as well as those in AE-L task for BERT(d), ELECTRA (e) and T5 (f).

Supplementary Figure S3. Percentages of AE signals by category in all AE-containing articles

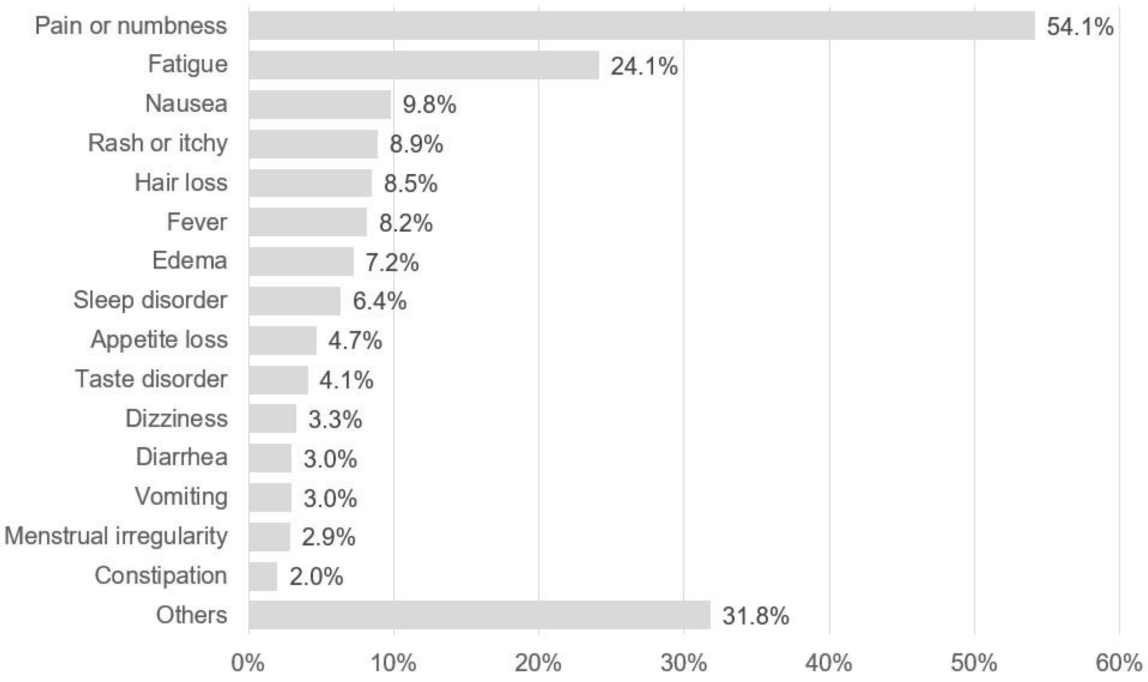

The percentage was calculated by dividing articles with all AE signals in each category by all AE-containing articles (i.e., 893 articles)

Supplementary Figure S4. Distribution of token numbers in the 2,272 blog posts written by breast cancer patients

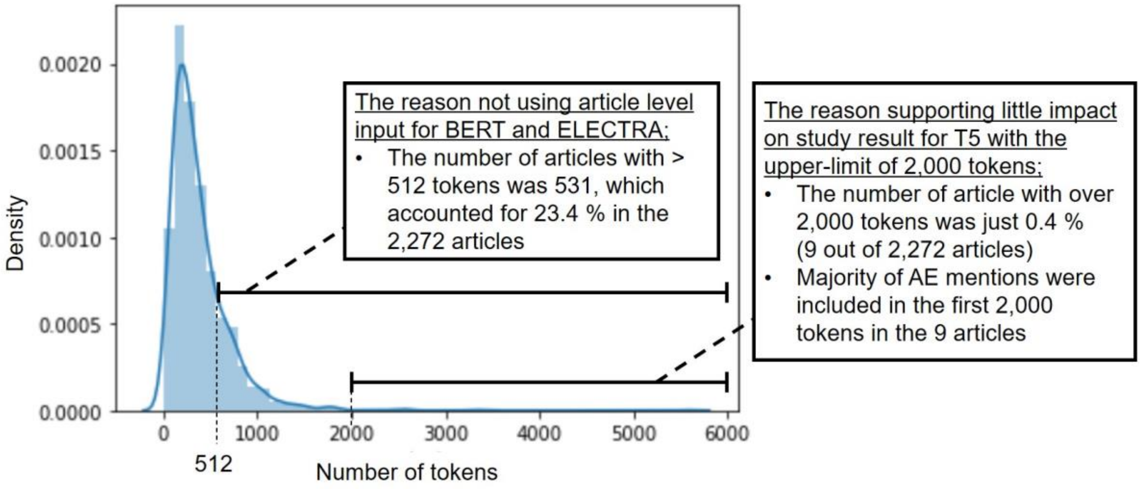

Supplement: Supplementary file 1 — Supplementary Information. [file 41598_2023_42496_MOESM1_ESM.pdf]
